# To form and function: on the role of basement membrane mechanics in tissue development, homeostasis and disease

**DOI:** 10.1098/rsob.200360

**Published:** 2021-02-17

**Authors:** Nargess Khalilgharibi, Yanlan Mao

**Affiliations:** ^1^ MRC Laboratory for Molecular Cell Biology, University College London, Gower Street, London WC1E 6BT, UK; ^2^ Institute for the Physics of Living Systems, University College London, Gower Street, London WC1E 6BT, UK

**Keywords:** basement membrane, mechanics, tissue shape, tissue dynamics, basement membrane dynamics, basement membrane remodelling

## Abstract

The basement membrane (BM) is a special type of extracellular matrix that lines the basal side of epithelial and endothelial tissues. Functionally, the BM is important for providing physical and biochemical cues to the overlying cells, sculpting the tissue into its correct size and shape. In this review, we focus on recent studies that have unveiled the complex mechanical properties of the BM. We discuss how these properties can change during development, homeostasis and disease via different molecular mechanisms, and the subsequent impact on tissue form and function in a variety of organisms. We also explore how better characterization of BM mechanics can contribute to disease diagnosis and treatment, as well as development of better *in silico* and *in vitro* models that not only impact the fields of tissue engineering and regenerative medicine, but can also reduce the use of animals in research.

## Graphical abstract

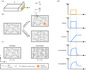

## 1. Introduction

Tissues must be of the correct size and shape in order to carry out their normal function. The basement membrane (BM) is a special type of extracellular matrix that lines the basal side of epithelial and endothelial cells and is important for the form and function of the overlying tissues. Exposure of the BM to mechanical stress is unavoidable and directly linked to BM function. Indeed, as a scaffold lining epithelial and endothelial tissues, the BM is constantly exposed to a variety of stresses, such as tissue growth during development or continuous deformations in normal physiology (e.g. blood flow, inflation/deflation of alveolae, movements of the gut). Despite the conceptual understanding of the network bonding and its response to stress, little is known about BM mechanics, especially rheology (box 1), and experimental measurements of BM mechanical properties, such as tensile strength and stiffness, have only recently emerged [1–3]. These measurements show that the BM is stiffer than the overlaying cells, suggesting that when a tissue is deformed, most of the applied stresses are borne by the BM [4,5]. Hence, the mechanical role of the BM in tissue sculpting and shape maintenance, on top of its biochemical and signalling roles [6–11], is becoming increasingly apparent.

Box 1. **Glossary of mechanical and biological terms.**

**Rheology:** Rheology is the study of how a material deforms and flows in response to an applied stress.
**Young's modulus:** Young's modulus is a mechanical property that quantifies the stiffness of a solid material (i.e. its resistance to deformation under an applied stress). A solid material with a higher Young's modulus is harder to deform. Young's modulus of a material can be obtained from the slope of the linear regime of its stress–strain curve. However, due to experimental complications, it is not always possible to measure the ‘actual’ Young's modulus, and some studies report an ‘apparent’ Young's modulus instead. Therefore, in this review, we use the general term of stiffness measurements to report all mechanical measurements of Young's modulus.
**Ultimate tensile strength:** The maximum tensile stress that a solid material can bear before breaking.
**Turnover rate:** The turnover rate of BM proteins quantifies how fast they are replaced within the BM and is often dictated by the deposition and degradation rates of the protein. If the BM proteins are produced locally by the tissue itself, then the deposition rate is limited by the rate of protein synthesis. However, if the BM proteins are produced externally and then transported to the tissue of interest, the deposition rate is limited by the rate of incorporation of the protein into the BM.
**Remodelling:** BM remodelling and turnover have often been used interchangeably in the literature. In this review, the remodelling refers to restructuring and reorganizing of existing BM components, i.e. without the need to synthesize/degrade material.
**Elasticity:** Elasticity represents the ability of a material to instantaneously deform to a time-independent strain when exposed to an external stress, and to return to its original shape when the external stress is removed. In a simple linear elastic solid, the stress (*σ*) and strain (*ε*) are proportional through a constant Young's modulus (*E*), i.e. $\sigma = E\; \times\; \varepsilon $.
**Viscosity:** Viscosity represents the resistance of a fluid to deform when exposed to an external stress, i.e. higher viscosity means higher resistance to flow and, therefore, slower deformation. In a simple Newtonian fluid (i.e. ideal viscous fluid), the stress (*σ*) and the strain rate (d*ε/*d*t*) are linearly proportional through a constant viscosity (*η*), i.e. $\sigma = \; \eta \times ( {\rm d}\varepsilon {\rm /d}t) $. Although no real fluid behaves exactly as a Newtonian fluid, some like water can be assumed to be a Newtonian fluid under normal conditions encountered in daily life.
**Plasticity:** Plasticity represents the ability of a material to deform non-reversibly and permanently when exposed to an external stress. Above a certain stress/strain threshold, even an elastic material may undergo a plastic deformation.
**Viscoelasticity:** Viscoelasticity describes the mechanical response of a material that exhibits both elastic and viscous behaviours. Viscoelastic material have three common features: stress relaxation (i.e. ability to relax the stress when exposed to a maintained step strain), creep (i.e. ability to continuously deform when exposed to a maintained step stress) and hysteresis (i.e. when exposed to cyclic loading, the stress–strain curves of loading and unloading are different). When the elastic response of a viscoelastic material dominates its viscous response, it is said that the material is more ‘solid-like’. Conversely, when the viscous response dominates the elastic response, the material is more ‘fluid-like’.
**Viscoplasticity:** Viscoplasticity describes the mechanical response of a material that undergoes a time-dependent irreversible deformation when exposed to external stress.
**Poroelasticity:** Poroelasticity describes the viscoelastic response of a biphasic material consisting of a porous solid phase and a fluid phase. The fluid pressure in the pores can contribute to the total stress of the material and can strain the material. Differential pore fluid pressure can result in fluid movement within the material. In tissues, upon an external strain, deformation of the porous medium generally pushes and redistributes the fluid within the porous solid phase.
**Mechanical anisotropy:** In a mechanically anisotropic material, the mechanical properties depend on the direction of measurement. An example of this is stiffness anisotropy, where the material is stiffer in a particular direction.

The BM is not a static structure, but a dynamic one that can change, either through protein synthesis/degradation or reorganization of its existing components [12]. These changes often lead to alterations in other features of the BM such as thickness and mechanical properties. While the dynamic nature of the BM is essential for tissue development, homeostasis and repair, dysregulated BM can be the cause of disease or contribute to its progression [13–17].

The BM has multiple roles in regulating how cells perceive biochemical signals, such as via integrin signalling, and through regulating ligand availability [6,18–20]. However, in this review, we focus on the mechanical role of the BM and its impact on tissue form and function. First, we provide a brief general description of BM composition and structure, and how they define the different mechanical properties of the BM. Here, it should be noted that despite some generic composition and mechanical properties of the BM, there are specific molecular, physical, spatial and temporal differences in the BM in different tissues. Next, we discuss how these properties can change during development, homeostasis and disease. In particular, we discuss the roles of mechanical anisotropy, as well as changes in BM stiffness, turnover and remodelling in defining and maintaining tissue shape. Considering the close connection between mechanics, shape and function, we also discuss how changes in BM mechanics due to ageing and disease affect tissue function. Finally, we explore the future perspectives and how advances in genetics, imaging and mechanical testing techniques can be employed to better characterize the BM mechanics, which can subsequently impact the fields of tissue engineering and regenerative medicine, and contribute to disease diagnosis and treatment.

## 2. Basement membrane composition and mechanics

The composition of the BM is highly conserved across metazoa, with its main constituents being: laminin, collagen IV, the glycoprotein nidogen and the heparan sulfate proteoglycan perlecan and/or agrin [6,21].

Laminins and collagen IV form two independent networks that are linked through proteins such as nidogen and perlecan [15,22]. Laminin is a heterotrimer of α, β and γ subunits that assemble into either a cross, Y or rod shape, with one long arm and a maximum of three short arms [22]. The laminin network forms through interactions of the short arms as sites of polymerization [15]. Collagen IV has a triple helical structure and forms a network through covalent dimerization of the C-termini and tetramerization of the N-termini, and non-covalent lateral associations of the triple helices [6,15].

To initiate the BM assembly, the long arm of laminin binds directly to the cell surface through integrins, α-dystroglycan and sulfated glycolipids [6,15]. This allows recruitment of other BM components nidogen, collagen IV, perlecan and agrin [6] in a temporal hierarchy [23,24]. A recent study in *Caenorhabitis elegans* showed that a poorly studied glycoprotein papilin, together with laminin and nidogen, is also involved in forming an initial BM network by the end of gastrulation, which was later complemented with collagen IV [24].

**Figure 1.**
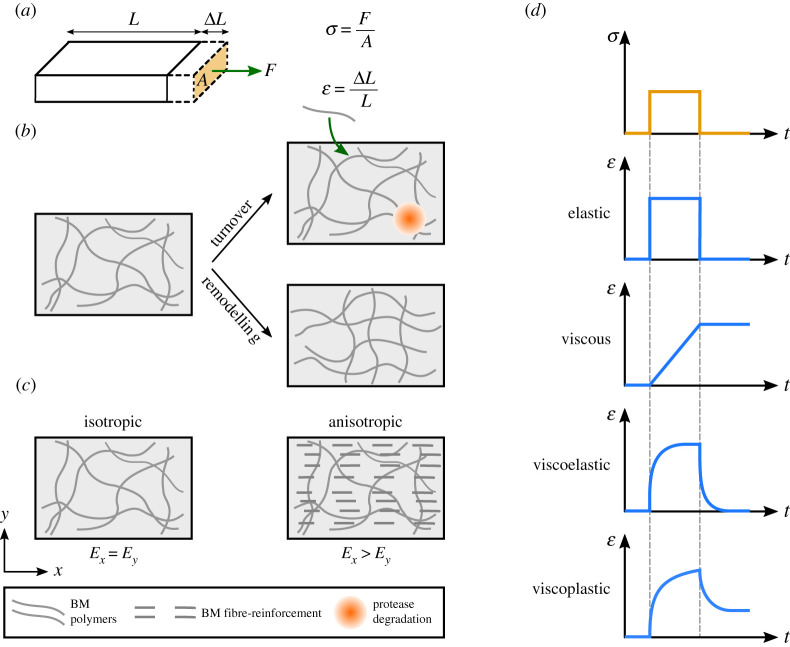
Basement membrane as a polymer network: schematic definition of the mechanical terms. (*a*) Stress *σ* is defined as force *F* per cross-section area *A*. Strain *ε* is defined as the change in length $\Delta L$ divided by the initial length *L*. (*b*) Turnover of a BM polymer network occurs due to the continuous incorporation of new material into the network (green arrow) and protease degradation (orange circle) of the existing material. In addition, the existing polymers can rearrange and remodel the BM polymer network. (*c*) The left panel demonstrates an isotropic material, where stiffness is the same in all directions. The right panel demonstrates an anisotropic material, where stiffness differs in different directions. The anisotropy can be achieved by polarized deposition of BM fibrils through fibre reinforcement. (*d*) The strain *ε* response (blue curves) of different material (i.e. elastic, viscous, viscoelastic and viscoelastic) subjected to a temporary step stress *σ* (orange curve).

Depending on the tissue type, the BM proteins are either secreted by the cells themselves or are synthesized by other tissues and then transported through body fluids to the site of assembly [11]. The cells also secrete proteases to degrade the BM. Together, these provide the BM with the ability to turn over and renew its structure during development and throughout life (box 1 and figure 1*b*). However, unlike many cytoskeletal structures that have a fast turnover (e.g. half-lives of tens of seconds for actin and myosin) [25,26], the rate of BM turnover (box 1) is widely thought to be slower, with reported half-lives varying from hours to months [27–33].

Water is a significant constituent of the BM [3,34]. Therefore, the BM can be considered as a biphasic material consisting of a porous solid phase (i.e. BM network) and a fluid phase (i.e. water). Upon exposure to mechanical stress, water can redistribute in the porous BM network, giving rise to a poroelastic (box 1) behaviour that dissipates the stress [34]. While poroelastic [35,36] and in general viscoelastic (box 1) behaviours [37–39] of synthetic extracellular matrices (i.e. hydrogels) have been widely studied, similar studies on natural BMs are limited [34]. It should also be noted that the rate of stress relaxation in poroelastic materials depends on the extent of deformation. Indeed, while studies on hydrogels and recently on natural BMs isolated from spheroids of human mammary epithelial cells (MCF-10A) have reported fast responses (i.e. sub-second to second time scales) for micrometre-sized deformations [34,36], the relaxation times of hydrogels can increase to tens of minutes to hour time scales for millimetre-size deformations [35,36]. Considering that different tissues undergo different extents of deformation during development and in normal physiology, further investigation is required to characterize the poroelastic behaviour of BMs and the time scales and extent of its contribution to tissue mechanics.

In addition to the extent of deformation that affects the poroelastic behaviour of BMs, the time scales of stress application also affect the overall mechanical response of the BM. At minute to hour time scales, BMs deform elastically (box 1) in response to external stress. This can be attributed to the slow turnover of the BM network and stability of its covalent bonds. Indeed, the covalent bonds of collagen IV network are thought to provide the BM with a solid-like behaviour [40] and mechanical stability [14,41]. At longer time scales, the non-covalent bonds of collagen IV, as well as other weak bonds in the BM (e.g. the interactions between laminin short arms to form a network), can break through exposure to mechanical stress, allowing the network to flow like a viscous fluid (box 1) and further dissipate stress [40,42]. Subsequent rebinding of these weak bonds will result in BM remodelling (box 1 and figure 1*b*) and plastic deformation (box 1) [40,42].

As discussed above, the complexity of the BM structure gives rise to its various time-dependent mechanical features, i.e. viscoelasticity and viscoplasticity (box 1 and figure 1*d*). Other mechanical properties such as stiffness (i.e. Young's modulus) and ultimate tensile strength (box 1) are also dictated by the composition and organization of BM components. It is worth noting that a change in BM composition or organization can affect more than one of its mechanical properties. For example, a change in BM composition can affect both stiffness and ultimate tensile strength, or BM remodelling can give rise to plasticity, while also affecting the network pore size and, therefore, poroelastic behaviour. In the following sections, we will discuss how the mechanical properties of the BM can change via different molecular mechanisms, and how these changes affect tissue form and function during development, homeostasis and disease.

## 3. Mechanical anisotropy: polarized fibril deposition

Mechanical anisotropy (box 1 and figure 1*c*) can be a beneficial property for the BM and its role in shaping tissues. Indeed, for a growing epithelium, being surrounded by a BM with anisotropic stiffness means that expansion is harder in one direction, which may then lead to oriented expansion, and therefore tissue elongation. As mentioned earlier, the BM is a specific type of extracellular matrix (ECM), the non-cellular protein structure that surrounds the cells and tissues in the body. In an ECM such as those of soft tissues, anisotropic stiffness can be achieved through arrangement of the fibrillar components (often collagen I) into parallel fibres [43,44], a phenomenon that is more generally known as ‘fibre reinforcement’ [45]. Fibre-reinforced materials exhibit higher stiffness parallel to the fibre alignment. However, unlike the ECM of soft tissues, the BM consists of non-fibrillar collagen IV that often forms an irregular isotropic mesh [46,47]. There are, however, a few examples where the BM is mechanically anisotropic [48].

**Figure 2.**
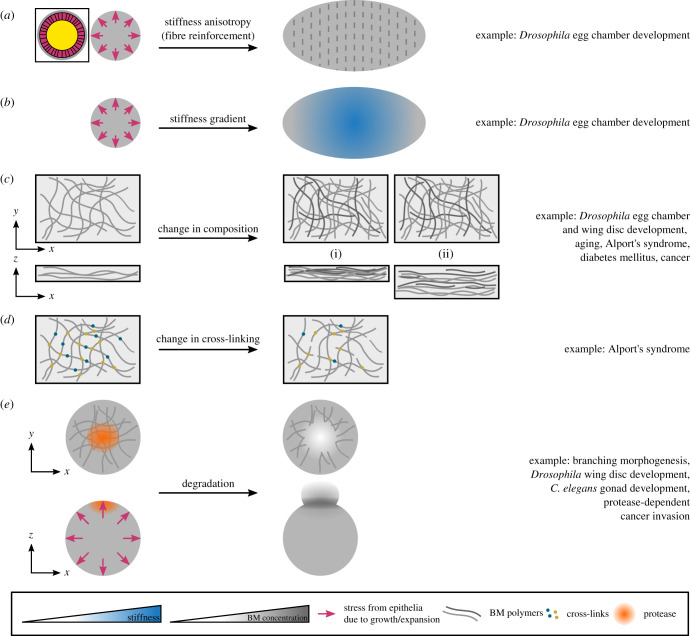
Mechanical anisotropy and changes in BM stiffness affect tissue shape. (*a,b*) A spherical tissue undergoing uniform growth/expansion can elongate through different mechanisms: (*a*) by creating a stiffness anisotropy and/or (*b*) by creating a stiffness gradient. The schematic in the box shows the cross-section of the spherical tissue consisting of BM (grey), epithelia (pink) and a central region (yellow) that is either a lumen (e.g. in spheroids) or is filled by cells (e.g. in the *Drosophila* egg chamber). (*c*) A change in BM composition can occur through a change in the levels of existing BM components or synthesis of additional components (darker lines in the right panels). Two scenarios can happen: the new material can increase the density of the network and keep the thickness constant (i), or increase the thickness of the network without changing density (ii). (*d*) A change in BM cross-linking can affect the connectivity of the network. (*e*) A uniformly growing spherical tissue is sculpted into a specific shape through local BM degradation. Local protease activity (orange circle) facilitates local bud formation and expansion.

**Figure 3.**
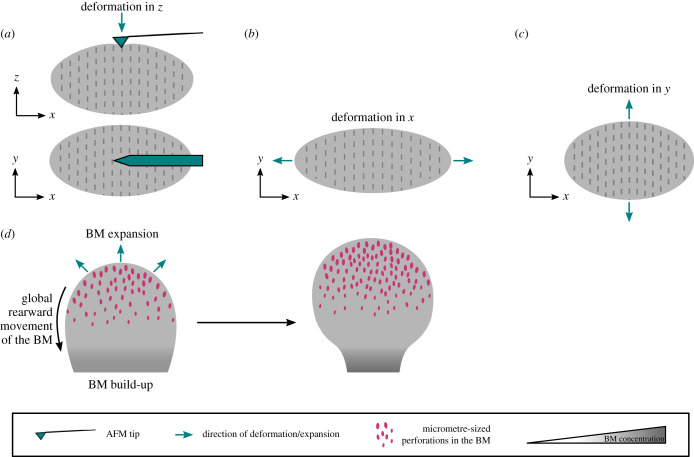
Specific examples of changes in the BM structure that affect tissue mechanics and shape. (*a–c*) Polarized fibril deposition in the BM of the *Drosophila* egg chamber is thought to create mechanical anisotropy in the tissue. (*a*) AFM measurements have shown that fibrils are locally more stiff than their surrounding BM [1]. However, these measurements are conducted perpendicular to the plane of the tissue (i.e. along the *z*-axis). (*b,c*) Using techniques such as uniaxial stretching [25,56], one can investigate whether the fibrils are creating mechanical anisotropy in the plane of the tissue. For example, if the stiffness measured along the *x*-axis (*b*) is smaller than the stiffness measured along the *y*-axis (*c*), the egg chamber will be mechanically anisotropic, which could be due to the presence of the fibrillar corset. The examples in (*b,c*) measure the global anisotropy in the stiffness. For more local measurements, techniques such as magnetic tweezers [57] can be used. (*d*) Morphogenesis of a single bud of the mouse salivary gland is illustrated. Appearance of hundreds of micrometre-sized perforations at the tip of the bud facilitate BM and tissue expansion, while global rearward translocation of the BM and its subsequent build-up towards the centre of the bud constrains expansion at the region and stabilizes the duct. This process has been described in [58].

The *Drosophila* egg chamber (follicle) is an interesting example where the tissue employs a fibre reinforcement mechanism to create anisotropy in the BM, which is then thought to guide morphogenesis. The egg chamber consists of 16 germline cells surrounded by an epithelial monolayer of follicle cells that is lined by a layer of BM [49]. It starts with a spherical morphology (i.e. aspect ratio of 1), but at the end of the 14-stage development, which lasts more than 3 days, it grows approximately 1000-fold in volume and reaches an aspect ratio of approximately 2.5 along the anterior–posterior (AP) axis [49]. From stage 1 to stage 9, the egg chamber rotates around the AP axis [48,50], a process that so far has been inseparable from elongation [51]. This tissue rotation is necessary for fibre reinforcement (figure 2*a*), whereby BM fibrils are deposited into the planar BM perpendicular to the axis of elongation [48,50,52,53]. It has recently been shown that a small GTPase Rab10 promotes secretion of the BM components into the basal pericellular space between the cells. These accumulated BM proteins will then get deposited circumferentially as the tissue rotates [54], leading to formation of a ‘fibrillar corset’ that is thought to constrain growth perpendicular to the AP axis resulting in tissue elongation [53]. In *C. elegans* embryos, a combination of mechanical measurements and computational modelling have shown that a cytoskeletal molecular corset creates an anisotropic stiffness to drive elongation [55], suggesting that a similar effect may arise from the BM corset in the egg chamber. Atomic force microscopy (AFM) measurements on egg chambers have shown that fibrils are more stiff than their surrounding BM, indicating that they can increase the stiffness locally [1]. However, these measurements were conducted perpendicular to the BM and it is yet to be determined whether polarized fibril deposition in the egg chamber creates anisotropic stiffness in the plane of the BM [51] (figure 3*a–c*).

It should be noted that although global polarized fibril deposition is dependent on tissue rotation, small levels of fibril alignment were observed even when the tissue rotation was blocked [54,59]. This is perhaps due to contractile forces exerted on the BM by the basal actin bundles that also align circumferentially [50,60]. However, it is not clear how much of this local remodelling and alignment contributes to BM anisotropy in normal conditions.

While egg chamber rotation is necessary for polarized fibril deposition that leads to a change in BM organization, the presence of BM is in turn necessary for tissue rotation. Indeed, mutations of the collagen IV α_2_ subunit (*viking* in *Drosophila*) and integrin β_PS_ subunit (*myospheroid*) perturb the polarized rotation and result in rounder eggs [48]. Furthermore, the speed of rotation is dependent on cell–BM adhesion [61,62] and the balance between laminin and integrin levels, which also dictates the time of onset of rotation [62]. Interestingly, fibril deposition occurs from stage 5 and the speed of rotation increases from stage 6 [50], suggesting a positive feedback mechanism whereby tissue rotation contributes to fibril deposition, which may then increase the speed of rotation [63].

By persisting after stage 12, the BM fibrils are thought to provide cues for reorientation of actin stress fibres via the dystroglycan–dystrophin complex, to drive further elongation [64]. From stage 13 and once elongation is complete, the molecular corset of BM fibrils is required to maintain elongation [48]. Interestingly, the ratio between the BM fibrils and the rest of the BM proteins forming the isotropic planar BM has been shown to be important for maintaining the elongated shape of the egg chamber. Indeed, producing too much BM fibrils (more than 37%) leads to egg chambers that elongate normally but cannot maintain their shape at later stages of development. This is perhaps because a smaller fraction of BM components is used to produce isotropic planar BM, which can then lead to its reduced density and weakening [54].

Finally, *in vitro* studies have provided evidence that a similar rotational motion may exist in other systems. For example, spheroids of human mammary epithelial cells (MCF-10A) rotate at a rate of 15–20 µm h^−1^ [65], comparable to that of the egg chamber [50]. Although it has been shown that this rotation is required for BM assembly [65], whether it leads to assembly of a structurally, and therefore mechanically, anisotropic BM is yet to be investigated.

## 4. Changes in basement membrane stiffness during development

In addition to fibril deposition that can induce anisotropy during development, other changes in the BM can create heterogeneities in its stiffness, affecting the BM resistance to the growth of its overlaying epithelia, and thus contributing to tissue sculpting. Here, we discuss some of these changes and how they affect tissue shape.

### 4.1. Establishment of stiffness gradients

BM stiffness gradients can act as global patterns to direct tissue expansion and morphogenesis (figure 2*b*). A well-studied example of this is again in the *Drosophila* egg chamber, where BM stiffness gradients are established at different stages of development, acting parallel to the mechanical anisotropy discussed above to drive tissue elongation.

AFM measurements have shown that during stages 3–6, the poles of the egg chamber are 50% softer than the central regions, with the difference becoming even larger at later stages [66]. Softening of the poles is regulated by JAK/STAT signalling. Interestingly, inhibition of this signalling pathway led to rounder egg chambers with a stiffness that was comparable to wild-type but homogeneous along the AP axis, demonstrating that the gradient and not the absolute values of stiffness is key for elongation [66]. This suggested that, by establishing a stiffness gradient, the BM was constraining the expansion at the central regions and facilitating it at the poles, therefore promoting elongation in a mechanism that likely acts parallel to the fibrillar corset mentioned above. These mechanical differences in the BM are then transduced through Src tyrosine kinase, affecting junctional E-cadherin dynamics leading to polarized cell-reorientation that further promotes tissue elongation [2]. Between stages 8 and 10, TGF-β activity leads to the establishment of another stiffness gradient, with the anterior becoming softer that the posterior [1]. This stiffness gradient, together with other spatial differences in the BM (i.e. denser and shorter fibrils at the anterior and thicker BM at the posterior), further contributes to tissue morphogenesis.

### 4.2. Changes in basement membrane composition

The levels of BM components change during development, affecting BM stiffness and therefore influencing tissue shape (figure 2*c*). Collagen IV is one of the main determinants of BM stiffness, with changes in its levels correlating with changes in BM stiffness. An example of this is during *Drosophila* egg chamber development, where collagen IV levels increase during stages 3–8 due to downregulation of the collagen-binding protein SPARC (Secreted Protein Acidic and Rich in Cysteine) [48,67]. Concomitantly, the BM stiffens [1,66]. Imaging stage 7/8 egg chambers has also shown that collagen IV has the same spatial pattern as the BM stiffness, with lower levels at the poles [66].

Other BM components also contribute to its stiffness, although their changes do not necessarily follow the same spatial and temporal patterns as collagen IV [62,66,67], highlighting their different effects on BM mechanics. Laminin seems to affect BM stiffness in the same way as collagen IV, as reducing laminin levels in the egg chambers resulted in lower collagen IV, and thicker but less compact BM that is also softer than wild-type BM from stage 5 [62]. The effect of perlecan on BM stiffness is more complicated, as both perlecan depletion [1] and overexpression [66] in egg chambers soften the BM. The reduction in BM stiffness following perlecan depletion may be due to structural defects in the BM, as depleting perlecan in *Drosophila* egg chambers and larval wing discs resulted in thin and fragile BMs [1,68]. However, in an intact BM, perlecan and collagen IV may have opposite effects on BM stiffness [66].

The opposition between perlecan and collagen IV is evident from their effects on tissue shape. Indeed, *Drosophila* wing discs depleted in perlecan are more compressed than wild-type, suggesting that perlecan counters the constricting force of collagen IV [68]. Similar opposing effects were observed in egg chambers, where perlecan overexpression and collagen IV depletion both inhibited elongation [48,66,67], although this may be due to the fact that both of these changes perturbed BM stiffness gradients [66].

Osmotic pressure experiments suggest that the effect of perlecan on other BM mechanical properties such as ultimate tensile strength is also complicated. In these experiments, tissues are immersed in water, which creates an osmotic stress on the BM due to the influx of water into the tissue. Wing discs depleted in perlecan broke more easily under osmotic pressure [68], suggesting that the ultimate tensile strength of the BM may also have been affected, although mechanical measurements are yet to be done to directly investigate this. Interestingly, but also contradictorily, egg chambers overexpressing perlecan also broke more easily under osmotic pressure [66]. Therefore, more in-depth studies are required to unravel the effect of perlecan on BM mechanics.

While the studies in *Drosophila* have primarily focused on the main BM components, a recent study in *C. elegans* gonad has shown that the less well-studied glycoprotein papilin affects BM composition to regulate BM expansion and allow for the extensive growth of the organ [24]. Indeed, papilin contributes to BM expansion by facilitating collagen IV removal, as depleting papilin led to accumulation of a fibrotic network of collagen IV and a 50% loss of surface area [24]. Considering the correlation between collagen IV levels and BM stiffness mentioned earlier, collagen IV removal through papilin may reduce BM stiffness, thus facilitating BM expansion due to organ growth.

### 4.3. The role of basement membrane cross-linking

In addition to collagen IV levels, covalent cross-linking of the collagen IV network is another determinant of BM stiffness (figure 2*d*). A study in *Drosophila* affected covalent sulfilimine cross-linking at the C-terminus of collagen IV by varying the bromine levels in the flies' diet [69]. Indeed, bromide, the anion form of bromine, is a cofactor of peroxidasin, an enzyme that catalyses formation of sulfilimine cross-links. The study showed that raising the flies in a bromine-deficient diet results in rounder eggs, while increasing the bromine levels to more than physiological conditions results in more elongated eggs [69]. Changing bromine levels did not affect the collagen IV levels, suggesting that changes in the egg aspect ratio may be due to changes in the BM mechanical properties (e.g. stiffness) affecting how well the BM can constrain the egg chamber circumferentially to drive elongation. Recently, direct mechanical testing of mouse renal tubules showed that reducing sulfilimine cross-linking leads to a reduction in BM stiffness [70].

Bromide-mediated sulfilimine cross-linking may also affect other mechanical properties of the BM. For example, the BM of the midgut of *Drosophila* larvae grown on a bromine-deficient diet were more diffuse, thicker and perforated [69], suggesting that ultimate tensile strength may be reduced due to lower BM integrity. Considering that the BM has several types of covalent cross-links, more detailed mechanical measurements are required to better characterise how they affect BM mechanics.

### 4.4. Basement membrane degradation

The confinement imposed by the BM is important for developing and maintaining tissue shape. An example of this is in *Drosophila* wing discs, epithelial sacs consisting of two epithelial layers (the peripodial and the columnar epithelia), that give rise to the animal's wing through metamorphosis. In early larval stages, the confinement imposed by the BM acts together with differential cell growth and apical constriction to initiate folding [71]. Furthermore, by physically constricting the cells, the BM helps to maintain the wing disc's folded morphology, as evidenced by cell flattening and tissue unfolding following BM degradation [68]. However, the confinement imposed by the BM (and therefore its stiffness) may also need to be modulated at times, to sculpt tissues into complex shapes. This can be achieved by spatially and temporally controlled BM degradation through expression of proteases.

Matrix metalloprotease (MMP)-mediated BM degradation has been shown to be essential for different stages of *Drosophila* wing development [72–75]. During L3 larval stage, local degradation of the BM of the columnar epithelium is necessary for fold progression [72,73]. Later, the degradation of the BMs of the peripodial epithelium and its neighbouring larval epidermis is required to remove the barrier between the two epithelia and allow for wing disc eversion [74]. The MMP-mediated BM degradation is also required for the columnar-to-cuboidal cell shape changes during pupal wing development [75]. The importance of BM degradation is particularly evident when comparing the wing and haltere discs (imaginal discs that give rise to halteres, end-knob-shaped organs that, together with wings, are necessary for flight [76]). Haltere discs are similar in shape but smaller than wing discs at late larval stages. However, their shapes start to differ in early pupal stages due to a delay in the haltere BM degradation. Indeed, in the haltere, the Hox gene Ultrabithorax downregulates MMP1, delaying collagen IV degradation and therefore affecting further cell shape changes [76]. This prevents tissue expansion and apposition of dorsal and ventral regions [76], giving rise to the morphological difference between these two organs within a few hours [75,76]. BM degradation also occurs in the *Drosophila* leg disc, although a recent study has shown that unlike the wing disc, it is not essential for the opening and retraction of the peripodial epithelium, but is still necessary for its eversion [77].

In branching organs, such as lungs, it has long been known that there are spatial differences in the BM at the tip of the expanding buds compared to more static regions of ducts and clefts (figure 2*e*). These differences include thinning, increased degradation and discontinuities in the BM [78–81]. The increased degradation and discontinuities at the tip [78] are thought to reduce BM stiffness and make it more compliant, which will then facilitate its local deformation by the cytoskeletal tension of the growing epithelia, resulting in BM expansion and thinning [82]. Indeed, reducing tension by inhibiting myosin II through ROCK inhibitor led to BM with homogeneous thickness in embryonic day (E) 12–14 mouse lungs [82]. The local changes in the BM also feedback through signalling pathways to affect cell growth, leading to higher proliferation at the tips [82], therefore further contributing to bud expansion.

The role of local BM degradation in tissue sculpting was studied in detail in the mouse salivary gland [58]. In this organ, around E13, when most of the expansion occurs, hundreds of micrometre-sized perforations form in the BM at the tip of the bud [58], making that region more compliant to expansion, therefore promoting branching. Concurrently, the BM as a whole moves rearward at a rate of 8 µm h^−1^ from the tip towards the centre where it builds up again, closing the perforations [58]. Mechanically, the BM build-up at the centre of the bud could increase the BM stiffness to constrain expansion at this region, stabilizing the ducts and further supporting branching (figure 3*d*). Both protease activity and myosin II contractility are necessary for the formation and maintenance of the perforations, as well as global rearward movement of the BM, because inhibition of either of these perturbed both processes [58]. Finally, bleb-like protrusions into the perforations were also observed, suggesting that the cells use these protrusions to punch into the BM and then use contractility to stretch the BM and translocate it rearward [58].

Recently, similar perforations were identified in early post-implantation (E5–6.5) mouse embryos [83]. These perforations were distributed evenly around the epiblast but then localized posteriorly after anterior visceral endoderm (AVE) migration, which defines the AP axis [83]. It was shown that the AVE inhibits Nodal activity on its underlying epiblast. MMP expression, which is regulated through Nodal signalling, was therefore also inhibited and subsequent formation of perforations only occurs at the posterior side of the embryo. Consistent with observations in mouse salivary gland [58], perforations orient in the direction of growth, suggesting that they ease confinement introduced by the BM to allow for tissue expansion [83]. Finally, perforations persist after the initiation of gastrulation, to allow for a local increase in BM compliance and therefore facilitate extension of the primitive streak [83].

## 5. Changes in basement membrane stiffness due to ageing

As the body ages, the BM changes in composition and structure, affecting its mechanical properties such as stiffness. Since withstanding external stresses is an important function of epithelia and their underlying BM, changes in BM stiffness can directly affect tissue functionality with age.

A study looking at the human inner limiting membrane (ILM), the BM in the boundary between the retina and the vitreous body showed an increase in collagen IV and agrin levels and a decrease in laminin levels with age [3]. The study also reported an increase in stiffness, which could be due to the increase in collage IV levels. A similar age-dependent increase in the collagen IV levels has been reported in the vascular BM of the brain [84]. Recently, an age-dependent lipid accumulation was reported in the BM of the blood–brain barrier [85], which may lead to further compositional changes in the BM, and contribute to neurodegeneration and BM thickening [85].

Indeed, BM thickening (figure 2*c*) is another age-dependent change that has been reported in multiple human tissues, such as the ILM [3], capillary BM [86], glomerular BM [87] and epidermal BM [88], as well as BM from other species such as mice, rats and gerbils [85,89,90]. The BM can thicken due to changes in composition and/or turnover rates (discussed in §7). In the human ILM, BM thickness starts at 70 nm in fetal stages, increasing to 300–35 nm at the age of 22 years and finally to a few micrometres at the age of 90 years [3]. Interestingly, as the ILM thickens, its stromal side facing the vitreous body remains smooth, while the epithelial side facing the retina becomes more irregular over time with indentations growing into the retina [3].

The difference between the stromal and epithelial sides of the BM has been extensively studied in the ILM and two other BMs of the adult human eye [91]. All three BMs rolled up after excision, with their epithelial side being the outer surface and their stromal side being the inner surface. This may be due to the higher number of cells and therefore cell–ECM binding sites on the epithelial side compared to the stromal side, which may then give rise to a higher compression on the epithelial side. Isolation of the BM from its neighbouring tissues upon excision removes this compression, leading to the expansion of the epithelial side and consequent rolling of the BM [91].

The stromal and epithelial sides of the three ocular BMs mentioned above also differed in stiffness, with the epithelial side being about two times stiffer than the stromal side [91]. Furthermore, there was a side-specific distribution of BM components, with laminin localizing to the epithelial side and the N-terminus of collagen IV localizing to the stromal side of all three BMs, while the localization of the C-terminus of collagen IV varied between the three BMs. In ILM and Descement's membrane, the BM separating the corneal endothelium and stroma, the C-terminus localized to the epithelial side, while in the lens capsule, it was found on both sides [91]. Considering the 400 nm length of collagen fibres [6], it is possible that the spatial separation between the collagen IV domains requires a minimum BM thickness, as a similar side-specific separation was not observed in BMs of other species with thinner BMs (less than 100 nm) [91]. It would therefore be interesting to investigate whether the age-dependent BM thickening allows for this spatial separation of collagen IV domains and whether this contributes to the mechanical and functional differences between the two sides.

Changes in hormonal levels also occur during ageing, such as a decrease in oestrogen levels following menopause, which can also affect the BM. Indeed, oestrogen can directly regulate protease activity [92–94]. A recent proteomic analysis on mouse skin has revealed that levels of BM proteins laminin and nidogen increase in oestrogen-deficient and aged skin, suggesting that oestrogen regulates the BM during ageing [95]. Interestingly, the study showed that hormonal changes and ageing have opposite effects on the ECM mechanical properties, with stiffness and ultimate tensile strength decreasing in hormone-deficient mice and increasing due to ageing [95]. It is yet to be understood how hormonal changes affect the composition, structure and mechanical properties of the BM, independent of ageing.

## 6. Changes in basement membrane stiffness due to disease

### 6.1. Alport's syndrome

There are a number of diseases, including genetic and autoimmune diseases, directly caused by dysregulation of BM components [15,96,97], leading to changes in mechanical properties of the BM and affecting its function. One of the major examples is Alport's syndrome, a genetic disease associated with mutations in collagen IV genes that encode for a specific ${\rm \alpha }_ 3 .{\rm \alpha }_ 4 .{\rm \alpha }_ 5$ (IV) collagen heterotrimer found in the kidney glomerular BM, as well as a limited number of other BMs [98,99]. During development, the more common ${\rm \alpha }_ 1 .{\rm \alpha }_ 1 .{\rm \alpha }_ 2$ (IV) collagen is partially replaced by the ${\rm \alpha }_ 3 .{\rm \alpha }_ 4 .{\rm \alpha }_5$ (IV) collagen network that has higher cross-linking and therefore more mechanical stability [6,98,99]. In Alport's syndrome patients, this developmental switch does not happen, leading to a glomerular BM entirely made of ${\rm \alpha }_ 1 .{\rm \alpha }_ 1 .{\rm \alpha }_ 2$ (IV) collagen network, which has disrupted pore size and is less stable [6,15,98,99]. This affects the filtration barrier function of the glomerular BM, leading to proteinuria, haematuria and progressive renal failure [6,98,99].

The stiffness of the BM may also be affected in Alport's syndrome [100]. Indeed, a 30% decrease in overall tissue stiffness was observed in mouse models of Alport's syndrome (Col4a3^−/−^), when glomerular injury was minimal in histopathology [100]. This suggests that mechanical changes may contribute to disease progression, as well as highlight their potential for early diagnosis of the disease. The lower stability and changes in network pore size in Alport's syndrome suggests that other mechanical features of the BM such as ultimate tensile strength or poroelastic relaxation may also be affected, although measurements are yet to be done to investigate this. Finally, although Alport's syndrome is primarily caused by changes in the collagen IV network, further dysregulations of laminins and MMPs have been reported in mouse and human models of the disease [99], which could further affect BM mechanics and function.

### 6.2. Diabetes mellitus

Diabetes mellitus is one of the best examples where a disease is not directly caused by the BM, but affects the BM (due to high glucose-induced changes in BM protein turnover; see §7). As such, many diabetes-related complications are associated with changes in the BM. While diabetes-associated hyperglycaemia can be controlled by taking medications, long-term complications of diabetes such as nephropathy, retinopathy, neuropathy and delayed wound healing, all related to changes in the BM, cannot be controlled.

An increase in collagen content (collagen IV and VI) and a decrease in laminin and proteoglycan content have been reported in diabetic human BMs [101,102]. In addition to changes in the levels of the main BM components, proteins not specific to BM such as fibronectin and tenascin have also been identified in diabetic human BMs [102,103].

The BM stiffness is also affected during diabetes. AFM measurements on the human ILM and the lens capsule have shown an increased BM stiffness after long-term diabetes [102,103]. Conversely, the outer surface of vascular BMs has been shown to soften as a result of diabetes, despite the increase in their collage IV levels [102]. Although collagen IV levels may not necessarily be indicative of stiffness, as evidenced by the fact that the eye capsule with higher collagen IV levels is softer than the ILM [102], an increase in collagen IV levels in the same BM often results in higher stiffness (as discussed in §4.2). Therefore, it is yet to be understood why the vascular BMs soften and whether the same softening pattern is observed in the inner (i.e. epithelial/endothelial) surface, which has been shown to be stiffer than the outer (i.e. stromal) surface in ocular BMs [91]. Furthermore, the contribution of BM mechanics to the complications arising from diabetes is also yet to be investigated.

### 6.3. Cancer

Cancer is an example where dysregulation of BM and ECM proteins in general can alter the mechanics of the environment, which can then feed back to affect disease progression. Indeed, multiple BM components, most importantly laminin, are known to be overexpressed by different cancer cells [104,105]. Tumour growth also relies on the incorporation of BM components in order to form new blood vessels [6,22]. In addition, proteomic analysis of mammary carcinomas has revealed that, while in poorly metastatic tumours, only stromal cells produce laminin and collagen IV, in highly metastatic tumours both tumour and stromal cells produce these BM proteins [106].

Changes in ECM composition due to cancer often result in stiffer matrices that can affect cell behaviours through signalling pathways and contribute to malignancy [107,108]. Malignant phenotypes can also be induced in non-malignant epithelial cells *in vitro* by culturing them in stiff matrices, which changes their chromatin state [109,110]. BM stiffening has also been shown to trigger prostate epithelial cell invasiveness in the ageing prostate gland [111]. Furthermore, metastatic tumours derived from the same primary tumour have been shown to create different ECM niches in different organs [112]. Finally, a recent study has unravelled the link between BM mechanics and tumour architecture and progression [113]. Indeed, it was shown that BM softening, together with an increase in the BM assembly (which affects BM turnover, discussed in §7), results in budding observed in pre-malignant basal cell carcinomas [113]. Conversely, BM stiffening leads to folding observed in invasive squamous cell carcinomas [113]. Therefore, it would be interesting to characterize how parameters such as the metastatic potential of tumours and their host organs affect the BM and ECM mechanics, and how this will then feed back to affect tumour architecture and disease progression, in a self-perpetuating cycle.

## 7. Basement membrane turnover

Studies aimed at characterizing the homeostatic turnover of the mammalian adult BMs have reported a range of numbers, from hours [28] to days [29,30], weeks [31,32] and months [33]. However, a recent study in the *Drosophila* embryo has shown a more rapid turnover of BM components with half-lives of approximately 7–10 h [27]. Interestingly, turnover rates were dependent on specific protease activity and the composition of BM. For example, collagen IV turnover was slower in MMP1 mutants but faster in nidogen mutants [27], consistent with suggestions of nidogen's role in stabilizing the BM [114].

The differences between the BM turnover rates of the *Drosophila* embryo and those of the adult BMs may be due to differences between species, as well as the fact that embryonic BMs may need to be more dynamic to accommodate for the extensive growth and deformations that tissues undergo. Indeed, in the ILM, a significant downregulation of BM protein synthesis was reported within the first 2 years of life [115], which may affect the turnover rate. It should be noted that the slow turnover of adult BMs, in combination with a relatively slower rate of degradation, could be one of the causes of BM accumulation and thickening observed during ageing [3]. Finally, the differences between the measurement techniques may also contribute to the variability between embryonic and adult BM turnover rates. Therefore, with the advances in microscopy techniques that enable long-term live imaging of fluorescence-labelled proteins, it is timely to revisit the homeostatic turnover of adult BMs. In particular, it would be interesting to compare the BM turnover rates in different organs and investigate whether they are affected by the organ's form and function, such as the extent and rate of deformations that tissues undergo.

**Figure 4.**
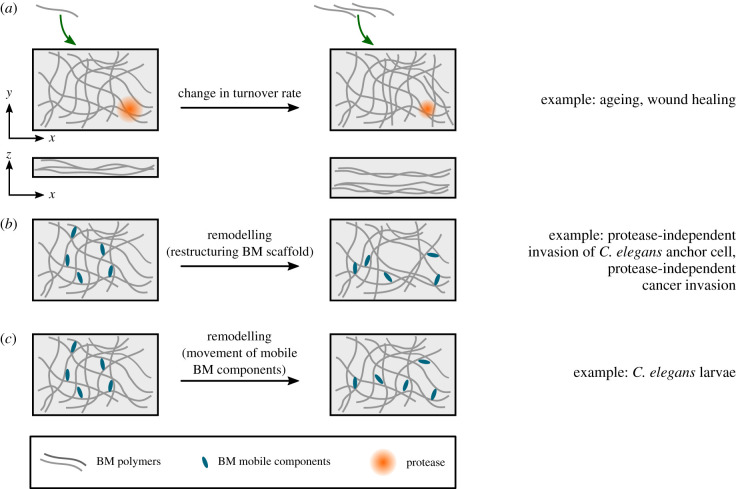
Change in turnover rate and remodelling of the BM affects its structure. (*a*) An increase in BM deposition and decrease in BM degradation affects the BM structure and leads to its thickening. (*b*) The BM scaffold can remodel through restructuring of its existing components. (*c*) Movement of mobile BM components through the static BM scaffold can also remodel the network.

BM turnover may be affected during disease. For example, an increase in the expression of TIMPs, inhibitors of metalloproteases and a decrease in MMPs expression have been reported in diabetic human BM [116]. These changes in expression levels may be due to changes in integrin expression in a high glucose environment, which can then affect regulation of proteases and BM components [116]. Changes in BM regulation and turnover can then lead to excess accumulation of BM (figure 4*a*). This, together with changes in BM composition, can be the cause of diabetes-induced BM thickening reported in many BMs, including the ILM and retinal vascular BMs [103], the glomerular and tubular BMs of the kidney [116] and vascular BMs [102].

Tissues and their underlying BM are prone to damage and injury throughout life. For example, while monocytes can squeeze through the existing gaps in the BM without affecting them, neutrophils expand the BM gaps, causing an inevitable but transient disruption to BM integrity [40,117]. Other examples include the damage to epidermal BM due to sun UV exposure [118] and skin injuries [119]. The increased protease levels in chronic wounds and pressure ulcers [120] may also affect BMs. Therefore, repairing the BM is an essential part of the wound healing process to ensure tissue integrity and maintain tissue shape.

Synthesis and turnover of BM components enables the BM to repair. Wounds in which the BM is affected have a slower healing rate, perhaps because of the slow turnover rate of BM components compared to their overlaying epithelium. Indeed, a study in rabbit cornea has shown that artificial wounds induced only on the epithelium can repair within days while wounds damaging both the epithelium and the BM require a minimum of six weeks in order to establish a tight adhesion between the epithelium, BM and stroma, and therefore fully repair [121]. In another study, when both the epithelium and BM were damaged, fibronectin and fibrin/fibrinogen were deposited within 8 h of wounding, providing a substrate for the epithelial cells to move on and close the wound within 2–3 days after wounding [122]. However, it took two to four weeks for the laminin and collagen IV to build up in the BM [122].

A study on the *Drosophila* L3 larval epidermis has shown that MMP1 is essential for BM repair and further re-epithelization during wound healing [123]. Indeed, while the majority of induced wounds closed within 18 h in wild-type animals, all of the wounds remained open in MMP1 mutants [123]. After wounding, upregulation of MMP1 through the jun N-terminal kinase (JNK) pathway led to accumulation of collagen IV around the wound within 5 h [123]. Interestingly, MMP1 was also localized to the wound edge, suggesting that it promoted the assembly of collagen IV, or its turnover, rather than degrading it [123]. Furthermore, overexpression of MMP1 increased the rate of wound healing [123]. Considering the effect of protease activity on BM turnover during development [27], it would be interesting to directly measure the effect of MMP1 on BM turnover during repair.

Another study on the *Drosophila* larval epidermis has shown that the hierarchy of BM reassembly during wound healing is different from its *de novo* assembly during development [124]. Most importantly, collagen IV recruitment was independent of laminin. In addition, scars remained on the BM 24 h after injury when the wound had closed [124]. In this study, it was not possible to investigate whether these scars would disappear at later stages, because the larvae underwent morphogenesis [124]. It would therefore be interesting to study the dynamics of this scarring in other systems and investigate how it changes the mechanical properties of the BM locally and whether this affects the BM and tissue function.

## 8. Basement membrane remodelling

As mentioned in §2, depending on the duration of applied stress, BMs may undergo elastic or plastic deformations. Stretching experiments on *Drosophila* larval wing discs have shown that after being exposed to sustained stretches for as long as 30 min, tissues can still retain their original shape when the stretch is released [25], pointing to the minute-to-hour time scale and elastic nature of these tissues. This is different from similar *in vitro* experiments on epithelial monolayers devoid of an extracellular matrix, where monolayers remodel their actomyosin cytoskeleton to adapt their shape to an applied stretch within a minute [26], suggesting that in the wing disc, it is the BM that is giving rise to its instantaneous elastic behaviour at minute-to-hour time scales. The minute-to-hour time scale and elastic behaviour of the BM may play an important role during development and in adult physiology, allowing the tissue to maintain its shape while being continuously exposed to internal and external deformations [125]. Interestingly, wing discs stretched for several hours lost their elastic behaviour [25], suggesting that the BM might deform plastically after being exposed to long stretches [40,42]. Indeed, *in vitro* experiments on Matrigel, the reconstituted BMs derived from Engelbreth–Holm–Swarm (EHS) mouse carcinoma have revealed that the weak non-covalent bonds of the BM network break when exposed to maintained stress/deformation, resulting in a plastic behaviour [42], allowing the BM to flow and further dissipate stress [40,42]. Furthermore, increasing the time scale of the applied stress/deformation increases the degree of plasticity of the BM [42]. However, it should be noted that the complex and poorly defined composition of Matrigel, as well as both in-batch and batch-to-batch variability in its biochemical and mechanical properties, may hinder reproducibility of results and have raised concerns on how accurately it captures the behaviour of BMs *in vivo* [126,127].

In addition to external stresses, cells can use their cytoskeletal machinery to deform and remodel their underlying BM. One example of this is in the uterine–vulval attachment of *C. elegans*, tissues that are initially separated by the two closely placed BMs of the gonad and epidermis. During larval development, a specialized uterine cell called the anchor cell invades through the BMs and initiates the attachment by generating multiple actin-rich protrusions called invadopodia to apply a pushing force of approximately 30 nN on the BM, deforming it approximately 1 µm [128]. Eventually, one to two of these structures manage to breach the BM. Although MMPs are secreted near the site of invasion [129], suggesting that they may be used by the invadopodia to weaken the BM and reduce its stiffness, it has been shown that even in the absence of MMPs, the anchor cell can still invade through the BM by forming a large ARP2/3-mediated actin protrusion that breaks into the BM [129]. Once the BM is breached, the *C. elegans* orthologue of netrin receptor, DCC, focuses F-actin regulators at the breach site, leading to the formation of a single large invasive protrusion that further expands into the BM within an hour and widens the hole at a rate of approximately 0.2 ± 0.06 µm^2^ min^−1^ [130]. This has been shown to occur through remodelling and pushing the BM aside, rather than degrading it [130] (figure 4*b*).

A recent *in vitro* study has reported that a similar mechanism is used by cancer cells for protease-independent invasion in highly plastic matrices [131]. In these matrices, cancer cells used invadopodia to apply cycles of protrusive and contractile forces to deform and push away the matrix, permanently expanding its pores in order to invade [131]. This protease-independent invasion was restricted by lowering BM plasticity through increasing covalent cross-linking in the matrix [132]. It should be noted that invadopodia are also used by cancer cells for protease-dependent BM degradation and invasion [40].

In addition to the remodelling associated with restructuring of the BM scaffold, it has recently been shown that mobile BM components can move through the immobile scaffold, contributing to BM dynamics [24] (figure 4*c*). Indeed, fluorescence recovery after photobleaching (FRAP) experiments on the pharynx of *C. elegans*, L4 larvae have shown that laminin and collagen IV form a stable immobile network with its components being turned over and replaced over the course of hours from extracellular resources (approx. 30% recovery in 5.5 h—comparable to the measurements in the *Drosophila* embryo) [24]. By contrast, other BM proteins such as nidogen and agrin were more dynamic (approx. 35–60% recovery in 15 min). The study showed that this higher rate of fluorescence recovery was due to the ability of these proteins to move through the stable laminin–collagen IV network at a speed of approximately 10–100 nm s^−1^ [24]. Furthermore, muscle paralysis significantly reduced this dynamic movement, showing that the animal's muscle contractions contributed to the mobility of BM components [24]. Further research is required to investigate whether similar BM mobility is present in embryonic and adult BMs in other species. In addition, considering the role of muscle contractions in the mobility of BM components, it would be interesting to see whether this mobility is significantly different in organs such as heart or lung that undergo continuous contraction/expansion. Finally, considering the cross-linking role of some of these mobile proteins, it is yet to be investigated whether their movement through the BM contributes to BM viscosity, and therefore time-dependent mechanical responses to stress/deformation.

## 9. Conclusion and future perspectives

For years, the BM was thought to be a static structure that provided physical support to tissues, as well as being a reservoir of biochemical cues. Recent findings are shedding light on the mechanical properties of the BMs (table 1) and how they change in development, homeostasis and disease.

**Table 1.** Examples of mechanical properties discussed in this review. For measurements of Young's modulus, it should be noted that some variability arises from the difference between the measurement techniques. In addition, as mentioned in box 1, some studies report ‘apparent’ Young's modulus, rather than the ‘actual’ Young's modulus, leading to more variability between the measurements. Finally, the stiffness of some material (including many biological tissues) is nonlinear, meaning that Young's modulus can be different at low and high strains. Therefore, where possible, we have provided the applied strain for clarification. To measure the degree of plasticity, a creep and recovery test is usually performed. This involves applying a constant stress to the material for a certain time and measuring the strain (creep test). Then the stress is removed and the strain is recorded until it reaches equilibrium (recovery test). The ratio between the final strain (i.e. strain at the end of the recovery test) to the strain at the end of the creep test is defined as the degree of plasticity. Since the extent and duration of the stress in the creep experiment affect the degree of plasticity in some material (including the BM), we have specified these values in the table.

material property	examples		measured values
Young's modulus	example BMs	*Drosophila* egg chamber	(stage 3) 30 kPa [1,66] (stage 7) 70 kPa [66], 250 kPa [1] (stage 8) 800 kPa [1]
		mouse renal tubules	(0–10% strain) 438 kPa [70] (30–40% strain) 3.23 MPa [70]
		adult human ILM	1.5–5 MPa [3]
		Matrigel	(8 mg ml^−1^, 1% strain) 100 Pa [132]
	other examples	human lung tissue	1.96 kPa [5]
		human skeletal muscle	5–170 kPa [5]
		human bone	10.4–20.7 GPa [5]
ultimate tensile strength	example BMs	cat lens capsule	1.7 MPa [133]
	other examples	mouse skin	(14 weeks old) 0.6 MPa [95]
		human bone	120–170 MPa [43]
		mild steel	500 MPa [43]
turnover half-life	example BMs	*Drosophila* embryo	7–10 h [27]
		rat glomerular BM	hours [28], days [29], months [33]
		mouse lung tissue	weeks [31]
		BM of mouse small intestine	weeks [32]
		human glomerular BM	days [30]
	other examples	F-actin	tens of seconds [25,26]
		myosin	tens of seconds [25,26]
		Keratin	hours [134]
degree of plasticity	example BM	Matrigel	(9.2 mg ml^−1^, 10 Pa stress maintained for 300 s) 0.4 [42] (8 mg ml^−1^, 10 Pa stress maintained for 1 h) 0.8 [131]
	other examples	mouse heart tissue	(1 kPa stress maintained for 300 s) 0.1–0.4 [42]
		mouse brain tissue	(1 kPa stress maintained for 300 s) 0.4–0.6 [42]
		polyacrylamide gels	(20–80 Pa stress maintained for 300 s) 0 [42] (100 Pa stress maintained for 1 h) 0 [131]
		silly putty	(100 Pa stress maintained for 1 h) 1 [131]

The best characterized mechanical property of BMs is its stiffness, which is often measured using AFM. However, AFM measurements are conducted perpendicular to the plane of the tissue, while stiffness in the plane of the tissue is likely what is sensed by the growing epithelia as they try to deform the BM (figure 3*a–c*). Techniques such as magnetic tweezers [57] and uniaxial stretchers [25,56] can be used to characterize the in-plane mechanical properties of BMs, although BMs may need to be isolated in order to be used with uniaxial stretchers. Brillouin microscopy is another novel technique that, due to its non-invasive nature, is providing great promise for *in vivo* characterization of spatial and temporal changes in BM stiffness during development and disease [135–137]. Another area that needs further characterization is time-dependent mechanical responses, such as viscoelasticity, for which all the above methods can be used.

Fluorescent tagging of proteins (e.g. CRISPR–Cas9-mediated tagging) have enabled the imaging of numerous BM components in *C. elegans* [24]. Combined with advances in microscopy techniques that allow long-term imaging of living samples, this will enable more accurate *in/ex vivo* characterization of BM thickness, structure and remodelling. The importance of conducting *in/ex vivo* live imaging experiments becomes clear when comparing the BM thickness measurements. Indeed, AFM measurements of BM thickness of unfixed and hydrated tissue have shown a two to four times larger values than more traditional transmission electron microscopy measurements [3,91]. Long-term imaging can also be combined with mathematical modelling to characterize the turnover of BM proteins [27]. In particular, the turnover of adult BM may need to be revisited using *in vivo* techniques, such as FRAP.

Computational models with an explicit implementation of BM have already shed light on how BM mechanics can affect tissue shape [71,138]. Better experimental characterization of changes in BM mechanics is beneficial for these models, as it will improve their parametrization and therefore predictive power. This will in turn allow computational models to be used to test how BM mechanics affects tissue shape in different scenarios *in silico*, allowing scientists to minimize the set of experiments conducted *in vivo*, therefore saving time and resources. In particular, this will help reduce the use of animals in research, since currently many *in vivo* experiments use animal models to study how the BM changes during development and disease. Computational models can also guide scientists to design extracellular matrix platforms with optimized mechanical properties for *in vitro* organoid growth and tissue engineering [126], a key step in engineering tissues with complex morphologies that is currently mainly done through trial and error [139]. This will ultimately allow scientists to replace many animal tissue models with their *in vitro* counterparts. Designing matrices with fine-tuned mechanical properties is also important for stem cell biology, where stem cell morphology, differentiation and behaviour are affected by BM mechanics [140].

Finally, changes in BM mechanics can be used as predictive measures for early diagnosis of disease [141]. Furthermore, understanding how BM changes during disease will allow clinicians to provide better therapeutic solutions to prevent/slow down the complications arising from changes in BM.

Going forwards, a better characterization of BM mechanics will be vital for our understanding of how tissue form and function is achieved during development, homeostasis and disease, and this will need the collective efforts of biologists, physics, engineers and computational scientists.

## Data accessibility

This article does not contain any additional data.
